# A minimal model for gene expression dynamics of bacterial type II toxin–antitoxin systems

**DOI:** 10.1038/s41598-021-98570-z

**Published:** 2021-09-30

**Authors:** Kosmas Kosmidis, Marc-Thorsten Hütt

**Affiliations:** 1 Physics Department, Aristotle University of Thessaloniki, 54124 Thessaloníki, Greece; 2 Department of Life Sciences and Chemistry, Jacobs University Bremen, 28759 Bremen, Germany

**Keywords:** Differential equations, Dynamical systems

## Abstract

Toxin–antitoxin (TA) modules are part of most bacteria’s regulatory machinery for stress responses and general aspects of their physiology. Due to the interplay of a long-lived toxin with a short-lived antitoxin, TA modules have also become systems of interest for mathematical modelling. Here we resort to previous modelling efforts and extract from these a minimal model of type II TA system dynamics on a timescale of hours, which can be used to describe time courses derived from gene expression data of TA pairs. We show that this model provides a good quantitative description of TA dynamics for the 11 TA pairs under investigation here, while simpler models do not. Our study brings together aspects of Biophysics with its focus on mathematical modelling and Computational Systems Biology with its focus on the quantitative interpretation of ’omics’ data. This mechanistic model serves as a generic transformation of time course information into kinetic parameters. The resulting parameter vector can, in turn, be mechanistically interpreted. We expect that TA pairs with similar mechanisms are characterized by similar vectors of kinetic parameters, allowing us to hypothesize on the mode of action for TA pairs still under discussion.

## Introduction

The vast majority of free-living bacteria contain a number of toxin–antitoxin (TA) gene pairs^1–4^. The toxin products target key cellular functions inhibiting cell growth and eventually leading to cell death, while the corresponding antitoxin neutralizes the toxin’s effect, thus, forming a TA system whose accurate expression regulation is vital to the survival of the cell^5^. These TA systems are currently classified in six groups (types I, II, III, IV, V, VI)^2^ according to the mechanism used by the antitoxin to neutralize the toxin. Types I-III are considered to be well-established TA systems^3,6–9^ while types IV-VI consist of newly discovered types^10–14^. Type II TA systems are the largest and best studied TA system class. Type II antitoxins are proteins. They typically have two domains, one that binds DNA and a second that binds and inhibits the activity of the cognate protein toxin^2,3,9^. The presence of TA systems is considered to be associated to persistence, i.e. the multidrug tolerance of bacteria, which obviously compromises the effectiveness of antibiotics on many pathogenic bacteria^15^. It is believed^4,15,16^ that when antibiotics are applied, a small sub-population of bacteria, called persisters, enters a dormant, non-dividing state and thus are protected from being killed. Experiments have shown a connection between persister formation and the competition between a toxin and its antitoxin inside an *E. coli* cell. Toxins inhibit cell growth and most antibiotics target the cell during the growth phase. Cells entering this persistent state seem to be immune to antibiotics but this immunity is different from the one obtained through advantageous mutations that result in antibiotic resistance since it is not permanent or inherited^17^. Knowledge about TA systems in bacteria is still accumulating^18^. This is true for the discovery of new TA modules^19^, their classification^5,20^, their functional roles^21–24^ as well as their detailed molecular mechanisms^25^. Very recently for example, it was discovered that the type II TA system *PrpT-PrpA* of the *Pseudoalteromonas rubra* plasmid, directly controls plasmid replication. It seems that the antitoxin *PrpA* binds to the iterons in the origin of replication (Ori), interfering with the binding of RepB to the Ori and, thus, preventing overreplication of the plasmid^26^.

**Figure 1:**
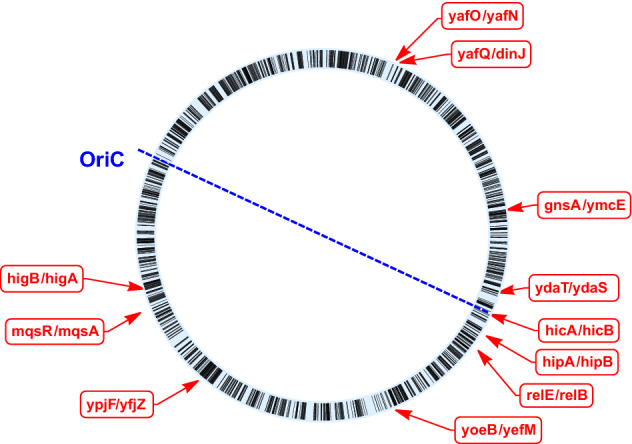
Genomic locations of the 11 TA modules studied in our investigation. Each gene is depicted as a radial dash on the circular chromosome. The location of each TA module is labelled with a red box. The *OriC*–*Ter* axis (from the origin of replication to the terminus of replication) is indicated for reference as a dashed blue line.

In *E. coli*, there are more than ten well-characterized type II TA systems^1^. These include *relE*-*relB*, *yafQ*-*dinJ*, *yoeB*-*yefM*, *hipA*-*hipB*, *yafO*-*yafN*, *hicA*-*hicB*, *higB*-*higA*, *ypjF*-*yfjZ*, *mqsR*-*mqsA*, *ymcE*-*gnsA* and *ydaT*-*ydaS*^10,27–37^. The genomic location of each of these TA systems is indicated in Fig. 1. It is of considerable practical importance to understand the dynamics of TA systems and several plausible models for TA dynamics and persister formation have been proposed (see, for example^38–40^ and references therein). It is also important that the proposed model predictions are compared to, nowadays available, high-throughput data. In this paper, we present a minimal model for the description of TA type II dynamics in *E. coli*. The basic characteristics of the minimal model is that it assumes: (a) regulation of toxin and antitoxin production rate by means of a negative feedback through DNA binding of the TA complex (b) toxin induced growth rate modulation. The model’s predictions are compared to the RNA-Seq gene expression data published in^41^ (see Results and Discussion).

TA dynamics have been of interest to mathematical modelling for a long time. So far, the focus of research has been on the basic dynamical properties of TA modules^39,40,42,43^ and the synchronization of multiple TA modules in response to environmental stimuli (e.g.,^44^), rather than the agreement with high-throughput data. For high-throughput data, in particular gene expression patterns, the dominant avenue of research has been to compare these patterns with large-scale regulatory networks or classes of regulatory mechanisms. In the case of bacterial gene regulation, successes have been understanding and experimentally confirming the role of small regulatory devices like feedforward loops^45,46^, the discovery of an interplay the regulatory network and chromosomal structure^47–50^ and the organization of gene expression along the axis from the origin (OriC) to the terminus (Ter) of replication^50^.

TA systems are often embedded in an intricate network of regulatory processes^5^ and part of functional regulatory modules^51^. There is evidence of collective behaviors arising from the interplay between TA systems. Such a model of coupled TA systems has for example been studied in^21^ and in^44^. Simple ordinary differential equation (ODE) models of (type-II) TA systems have for example been formulated in^21^ with an emphasis on coupled systems and the spontaneous switching occurring in stochastic dynamics, in^40^, where conditional cooperativity of the *RelBE* system has been studied and its response to environmental stimuli (e.g., nutritional stress), in^52^, which contains a simplified system capable of excitable dynamics, as well as in^39^ and^44^ with a focus on bistability. For type-I TA systems, a mathematical model has been developed in^53^, offering insight in time scales involved.

Here we study the long-term dynamics of TA pairs in time-resolved RNA-Seq data for *E. coli*. Our question is, whether the dynamics of all TA pairs in the data can be described by the same model, or whether qualitatively different models have to be assumed for the different TA modules.

## Methods

**Figure 2:**
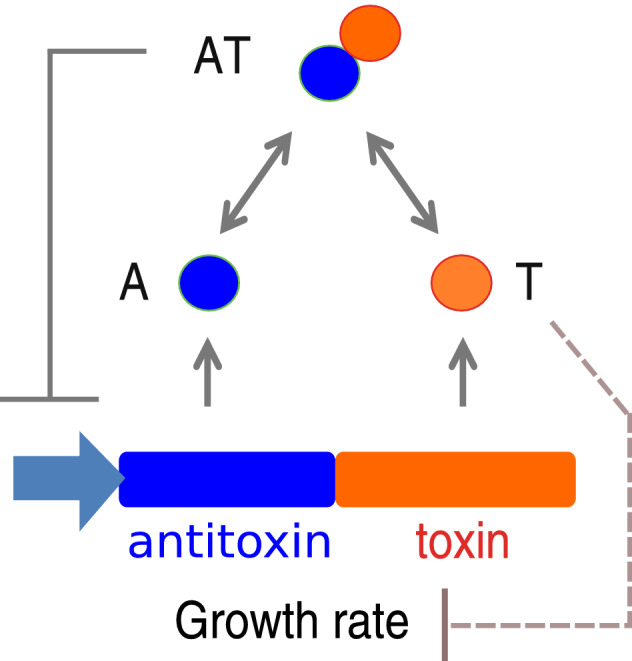
Schematic overview of the minimal model. Toxin *T* and antitoxin *A* are expressed by neighbouring genes. A TA complex molecule *AT* can be formed from one toxin and one antitoxin molecule. The *AT* molecule down-regulates *A* and *T* production. At the same time toxin molecules inhibit cell growth.

Figure 2 shows a schematic of the basic characteristics of the minimal model of type II TA gene expression. Toxin *T* and antitoxin *A* are expressed by neighbouring genes. It is known^1,39^ that toxins are more stable than the antitoxins, thus, the latter have to be constantly expressed in order to neutralize the toxin effects. The toxin and antitoxin form a complex *AT* which inhibits toxin and antitoxin production. More complex TA interaction (such as conditional cooperativity^39,40^ or cooperation between multiple TA systems^17^) are not included in the minimal model. Moreover, the presence of toxin has an inhibitory effect on the cell growth. This last fact is found to be an essential characteristic of an acceptable minimal model.

We denote the concentration of the antitoxin *A* with the variable $y_{1}$, that of the toxin *T* with $y_{2}$ and, finally, the concentration of the TA complex *AT* with $y_{3}$. The system of ordinary differential equations (ODEs) that describes the system is:

1 $$\begin{aligned} \frac{d y_1}{dt}&= \frac{k'_{1}}{\left( 1 + \frac{y_{3}}{s'_{1}}\right) \left( b'_{m} y_{2} + 1 \right) } - d_{1} y_{1} + d_{3} y_{3} - k_{3} y_{1} y_{2} \end{aligned}$$

2 $$\begin{aligned} \frac{d y_{2}}{dt}&= \frac{k'_{2}}{\left( 1 + \frac{y_{3}}{s'_{2}}\right) \left( b'_{m} y_{2} + 1\right) } - \frac{d_{2} y_{2}}{b'_{c} y_{2} + 1} + d_{3} y_{3} - k_{3} y_{1} y_{2} \end{aligned}$$

3 $$\begin{aligned} \frac{d y_{3}}{dt}&= - d_{3} y_{3} + k_{3} y_{1} y_{2} . \end{aligned}$$

Equation (3) is a standard chemical kinetics equation. We assume that the production rate of the complex $y_3$ is proportional to the product of the concentrations of $y_1$ and $y_2$, thus the term $k_{3} y_{1} y_{2}$ where $k_3$ is the respective rate constant. We also assume that the complex degrades to its constituents *A* and *T* with a rate constant $d_3$. To be precise, the rate constants $d_1, d_2, d_3$ are considered to be a sum of 2 terms due to a. protein degradation (specific destruction by specialized proteins in the cell) and b. dilution (the reduction in concentration due to the increase of cell volume during growth)^54^. This is the standard way of dealing with cell growth in the mathematical modeling of bacterial gene expression and is adequate in steady-state models. However, in the context of this work, since the abundance of free toxin can directly affect growth rate (and thus dilution), dilution cannot be properly characterized using a fixed number. Thus, the above model and, for that matter all other models in the scientific literature we are aware of, do not fully considered the effect of bacterial growth.

The inhibitory action of the *AT* complex is modelled through the inclusion of negative feedback terms such as $k'_{1}/\left( 1 + \frac{y_{3}}{s'_{1}}\right)$ in Eq. (1). The existence of toxin *T* in the cell reduces all protein production and decreases protein dilution by decreasing cell growth. Thus, the toxin concentration will have an inhibitory impact on the production rates of toxin, antitoxin, and on the cellular growth rate. We introduce an inhibition factor $1/(b'_{m} y_{2} + 1 )$ in Eqs. (1)–(2). The parameter $b'_m$ represents the redaction of protein expression due to the presence of toxin molecules. We also assume that growth inhibition will influence the toxin degradation rate, and we introduce a factor $(b'_{c} y_{2} + 1)$ that modulates the toxin degradation rate in Eq. (2), while we assume that the degradation rate of the free antitoxin remains the same. This is in agreement with a recent finding from^55^ that importantly, although free antitoxin is readily degraded *in vivo*, antitoxin bound to toxin is protected from proteolysis, preventing release of active toxin.

However, Eqs. (1)–(3), if one includes the unknown initial conditions for the quantities $y_1, y_2, y_3$ at $t=0$, contain 13 adjustable parameters. Our aim is to estimate the model parameters using experimental RNA-Seq data obtained from^41^. These experimental data (10 data points for each toxin antitoxin pair) would render such an estimation problematic, since such a model is structurally unidentifiable^56^.

In order to reduce the number of adjustable parameters we rescale the unobserved variable $y_3$ by setting $y_3 = (k'_2/d_3)z_3$ and rescale the variables $y_1, y_2$ by the same factor $\beta = k'_2$, i.e. by setting $y_1 = k'_2 z_1$ and $y_2 = k'_2 z_2$. Thus, we arrive at a system of ODEs for the rescaled variables $z_1, z_2, z_3$ which is:

4 $$\begin{aligned} \frac{d z_1}{dt}&= - d_{1} z_{1} + \frac{k_{1}}{\left( 1 + \frac{z_{3}}{s_{1}}\right) \left( b_{m} z_{2} + 1\right) } - k_{2} z_{1} z_{2} + z_{3} \end{aligned}$$

5 $$\begin{aligned} \frac{d z_{2}}{dt}&= - \frac{d_{2} z_{2}}{b_{c} z_{2} + 1} - k_{2} z_{1} z_{2} + z_{3} + \frac{1}{\left( 1 + \frac{z_{3}}{s_{2}}\right) \left( b_{m} z_{2} + 1\right) } \end{aligned}$$

6 $$\begin{aligned} \frac{d z_{3}}{dt}&= - d_{3} \left( - k_{2} z_{1} z_{2} + z_{3}\right) , \end{aligned}$$

where the new kinetic constants are related the those in Eqs. (1)–(3) by the relations $k_1 = k'_1/k'_2, s_1 = d_3 s'_1/k'_2, b_m = b'_m k'_2, k_2 = k'_2 k_3, s_2 = d_3 s'_2/k'_2, b_c = b'_c k'_2$. Moreover, we assume that $z_1$ and $z_2$ at time $t = 0$ are equal to zero and allow the unobserved complex concentration $z_3(0)$ to be equal to a constant $c_0$ which will be determined from the fitting of the solution of Eqs. (4)–(6) to the data. Henceforth, we will refer to the above model (Eqs. (4)–(6)) as the Z-model. The model is essentially a rescaled version of the model proposed in^39,40^ with the additional assumption that the antitoxin bound to toxin is protected from proteolysis.

Our numerical investigations have shown that the Z-model (Eqs. (4)–(6)) is the simplest model able to represent the complete set of the experimental data that we have in our disposal with reasonable accuracy. Omission of any of the above basic ingredients of the model (e.g. setting $b_m$ and $b_c$ equal to zero) leads to plausible models, which may describe adequately the time evolution of the concentrations of some TA pairs, but fail to describe the expression of the entire set. It is obvious to the reader that the Z-model and its variants that we examine in this manuscript are deterministic models. We will not deal with the important topic of investigating a stochastic variant of the Z-model through a Monte Carlo approach based on the Gillespie algorithm. Our modeling decision is based on the fact that the RNA Seq data that we will use to fit the model parameters are not single cell sequencing data. As one can see in the detailed description of the experimental data used in this study, each RNA seq “read” represents multi-cell averages on a timescale of hours. Of course for single cell RNA seq experiments a stochastic modelling approach would be more appropriate although admittedly much more difficult. There is, however, important progress in the direction of using stochastic models and the inference of parameter values from noisy data, see for example^57^. Bulk RNA-Seq data have clear limitations regarding such mechanistic interpretations. When technology advances (see, e.g.^58^ for an important step in this direction) and time-resolved single cell experiments are readily available, we envision that repeating our analysis could provide further valuable insights. In this case, however, it is known that, on a single cell level, mRNA and protein concentrations do not correlate well^59^. Repeating our analysis on a single cell level would then require time-resolved proteomics data.

For our analysis we used experimental RNA-Seq data obtained from^41^ (GEO accession number: GSE65244). The RNA Seq data used here are for the wild-type(wt) strain and obtained after the culture growth in rich medium during the stationary phase. The system of Eqs. (4)–(6) was solved numerically with custom code written in Python using the scipy python module^60^. Fitting of the numerical solutions of the ODE’s was performed as part of the code using the Nelder-Mead minimization algorithm as implemented in scipy. Since the task of performing fits for all TA pairs and all model variants is quite demanding the code was parallelized using the dask.distributed python module. All numerical simulations were performed on a workstation equipped with 2 Intel Xeon Gold 6140 Processors (72 cpu cores in total).

## Results

**Figure 3:**
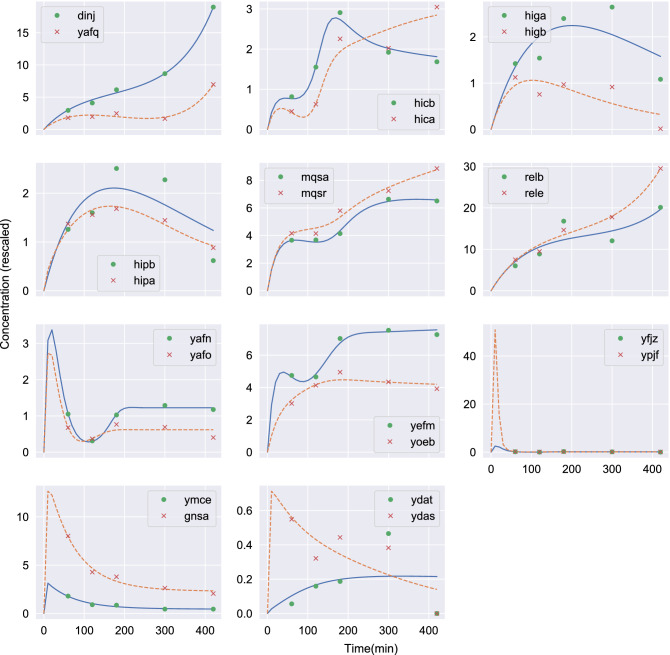
Toxin and antitoxin concentrations as a function of time for 11 known TA pairs of *E. coli*. Points represent RNA-Seq data for antitoxin (circles) and toxin (x-symbols) obtained from^41^. Solid lines show the result ($z_1(t)$) of the numerical solution of the ODE system, Eqs. (4)–(6) for the antitoxin. Dashed lines show the corresponding variable ($z_2(t)$) for the toxin.

Figure 3 shows the concentrations of toxin and antitoxin for 11 known TA pairs of *E. coli* as a function of time. Symbols represent experimental RNA-Seq data obtained from^41^ (GEO accession number: GSE65244). The above list is exhaustive meaning that it includes all the TA pairs for which there are experimental measures in the dataset. All data have been rescaled (multiplied by the same constant $c=10^{5}$ in order to avoid numerical errors during the fitting process). Lines are the numerical solutions of the ODE system, Eqs. (4)–(6). The kinetic constants of the system were estimated so that the weighted sum of the squared differences between the experimental data and the model predictions becomes minimum. We calculate weighted least squares since we have to fit two different experimental curves simultaneously whose *y*-axis values may differ considerably. Thus, we first calculate the mean values for each curve and then the weighted sum of the squared differences. Otherwise, curves with low mean values are practically ignored during the fitting process. Thus, the lines represent the “best” fit of the model to the data. We observe a very good agreement between the model predictions and the experimental data. As mentioned above, we assume that $z_1$ and $z_2$ at time $t = 0$ are equal to zero. This is a rather harsh, and possibly unrealistic, condition to impose. If more data points were available the more natural and appropriate choice would be to use the RNA seq measurements of the earliest available timepoint as our initial conditions. This is indeed the approach we took in our analysis in Appendix B (Supplementary Materials). We should point out, however, that since the same initial condition is imposed to all TA pairs and since there is no indication that the TA systems will exhibit chaotic dynamics—which is known to be rare in chemical systems, requiring rather special conditions—we do not have any reason to expect sensitivity of the dynamics to the initial conditions and, thus, we do not believe that our choice to affect the accuracy of the model. An additional analysis in Appendix B, where a different choice of initial conditions has been adopted, i.e. the average concentration across all measurements, seems to support such a claim.

**Figure 4:**
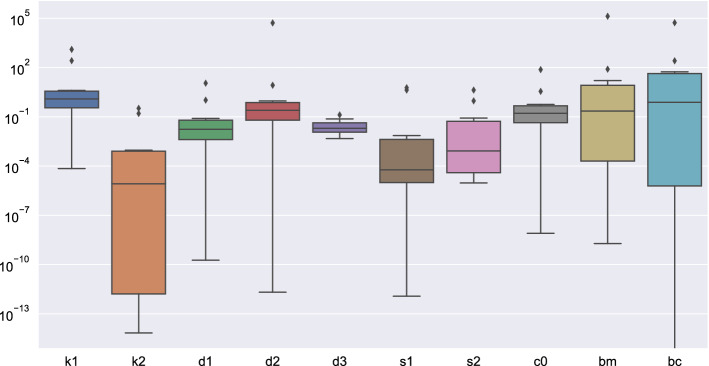
Box plot of the model parameters estimated from the best fit of the ODE system, Eqs. (4)–(6), to the RNA-Seq data. Each box shows the “dispersion” of eleven values, one per TA pair.

Figure 4 shows a box plot of the model parameters estimated from the best fit of the ODE system, Eqs. (4–6), to the RNA-Seq data. Each box shows the “dispersion” of eleven values, one per TA pair. We observe a wide distribution of parameter values across the different TA pairs. This is rather common in biological systems, where the kinetic constants of various metabolic reactions can differ by several orders of magnitude. Therefore, the same underlying differential equations lead to quite different dynamics precisely due to the broad range of the kinetic constants. In Appendix A we include a detailed discussion of the estimated covariances and standard deviations of the fitting parameters (see also the attached files in supplementary materials).

**Figure 5:**
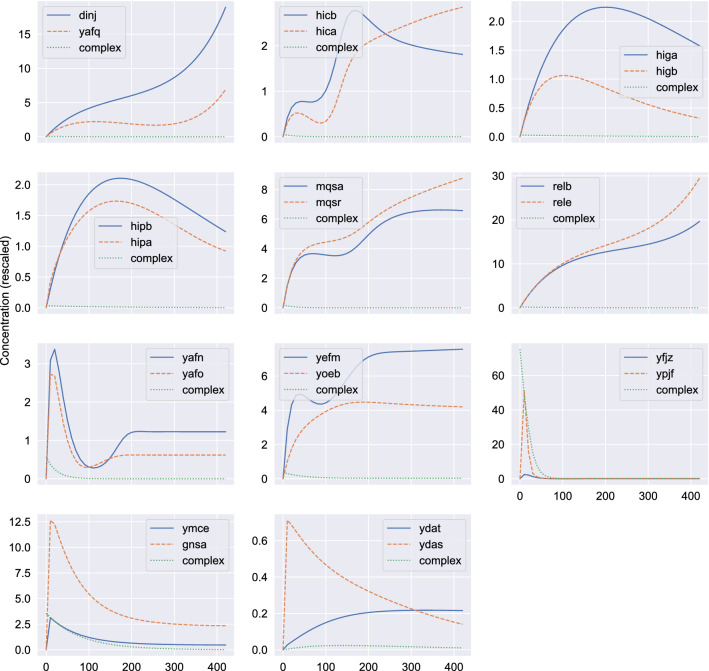
Toxin, antitoxin and TA complex concentrations as a function of time for 11 known TA pairs of *E.coli*. Solid lines show the result $z_1(t)$ of the numerical solution of the ODE system, Eqs. (4)–(6), for the antitoxin. Dashed lines show the corresponding variable $z_2(t)$ for the toxin. Dotted lines show the corresponding variable $z_3(t)$ for the TA complex. Notice that the *y*-axis has a logarithmic scale.

Figure 5 shows in a log-linear plot the toxin, antitoxin and TA complex concentrations as a function of time for the 11 known TA pairs of *E. coli*. Solid lines show the result $z_1(t)$ of the numerical solution of the ODE system, Eqs. (4)–(6), for the antitoxin. Dashed lines show the corresponding variable $z_2(t)$ for the toxin. Dotted lines show the corresponding variable $z_3(t)$ for the TA complex. We observe a variety of different dynamics, but interestingly enough in all cases the complex concentration $z_3$ seems to be lower than that of both the toxin and the antitoxin. For the majority of cases the antitoxin concentration is higher than that of the toxin. There are, however, exceptions, namely the *relB*-*relE*, *mqsR*-*mqsA* and the *ymcE*-*gnsA* pairs. The *ydaT*-*ydaS* pair also exhibits higher toxin expression for the most part of the observation time and only at the final stage the toxin level drop below that of the antitoxin. It is also quite intriguing that the Z-model predicts expression states where the toxin is constantly quite higher than the antitoxin (e.g. *ymcE*-*gnsA*) without resorting to the mechanism of conditional cooperativity^2,39^, although it is quite well-established that certain TA pairs (e.g. the *relB*-*relE* pair) exhibit conditional cooperativity and, obviously, such effects are not accounted for in the Z-model.

Next, we are interested in examining simpler versions of the proposed model and assessing their ability to describe the experimental data. We compare the Z-model to 7 simpler (i.e. with less adjustable parameters) variants, which we obtain from Eqs. (4)–(6) by forcing constraints on some of the constants, i.e. by fixing their numerical value or by setting them numerically equal to other constants. We describe these simpler variants below:

- Model “s1=s2” is obtained by forcing the constants $s_1$ and $s_2$ to have the same numerical value.
- Model “s1=s2 no bm” is obtained by forcing the constants $s_1$ and $s_2$ to have the same numerical value and by dropping the $b_m$ constant, i.e. setting $b_m = 0$.
- Model “s1=s2 no bc” is obtained forcing the constants $s_1$ and $s_2$ to have the same numerical value and by setting $b_c = 0$.
- Model “s1=s2 no bm bc” is obtained by forcing the constants $s_1$ and $s_2$ to have the same numerical value and by setting both $b_m =0$ and $b_c = 0$.
- Model “s1!=s2 no bm” is obtained by setting $b_m = 0$. Note that now constants $s_1$ and $s_2$ are allowed to have different numerical values.
- Model “s1!=s2 no bc” is obtained by setting $b_c = 0$.
- Model “no s1 s2 bm bc” is the simplest variant and is obtained from the Z-model ODEs by setting $s_1=1, s_2=1, b_m =0, b_c = 0$.

**Figure 6:**
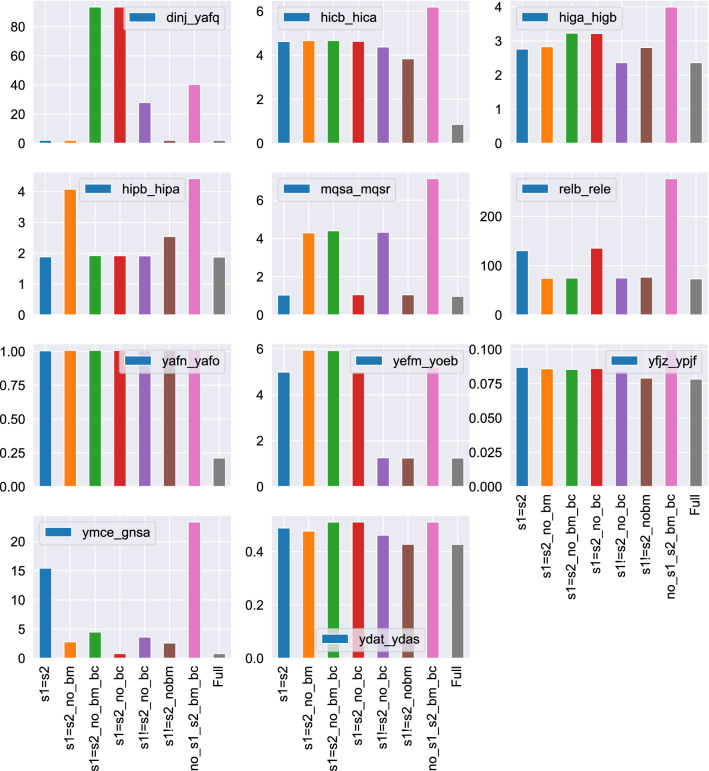
The values of the objective function for all TA pairs and for 7 model variants. The Z-model defined by Eqs. (4)–(6) is marked with the label “Full” in the x-axis.

Models, where the parameter $b_m$ is identically zero, do not take into account the reduction of protein expression due to the existence of toxin, while variants, where the parameter $b_c$ is identically zero, ignore the effect of growth inhibition. Figure 6 shows the minimum values of the objective function (i.e. the sum of weighted squared differences between model predictions and the experimental data) for all TA pairs and for the 7 model variants described above. The objective function values depend on the values of the experimental data which differ considerably between different TA pairs, thus the noticeable difference in the y-axis scales of Fig. 6.

The objective function of the Full Z-model is always lower than that of the variants, as expected. We should also mention that the algorithms (basinhopping in combination with a local Nelder-Mead algorithm) used for the minimization of the objective function are guaranteed to find local, not global, minima. Although we have performed a rather extensive search of the parameter space, there is always the chance that there are sets of parameters that will lead to lower values of the objective function than those reported here. We see that there are TA pairs for which simpler variants are capable of fitting the data with results comparable to those of the Full Z-model. However, the Full Z-model is the appropriate choice if one wants to describe the expression of the entire set of TA pairs.

**Figure 7:**
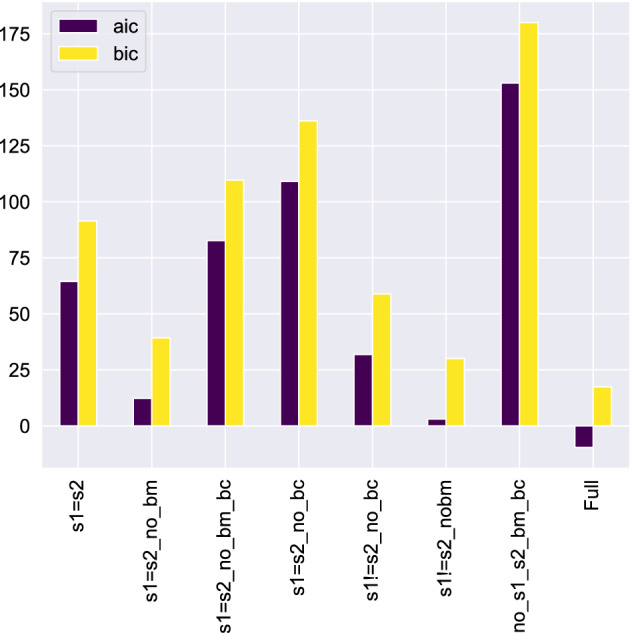
Collective Akaike Information Criterion (AIC) and Baeysian Information Criterion (BIC) for the Full Z-model and its seven variants. The Full Z-model has clearly the lowest AIC and BIC among all variants studied.

Since we want to compare models with different numbers of parameters, it might be plausible to examine two widely used model selection criteria, the Akaike Information Criterion (AIC) and the Bayesian Information Criterion (BIC) for the Full Z-model and its seven variants. These are calculated as follows:

7 $$\begin{aligned} \begin{array}{l} \text{ aic } =N \ln \left( \chi ^{2} / N\right) +2 N_{v} \\ \text{ bic } =N \ln \left( \chi ^{2} / N\right) +\ln (N) N_{v } , \end{array} \end{aligned}$$

where $\chi ^2$ stands for the sum of the squares of the residuals (i.e., the objective function discussed above), *N* is the number of data points (common for all model variants) and $N_{v}$ is the number of adjustable parameters for each model. $N_{v}$ is different for each variant. The full Z-model has the highest value, i.e. $N_{v} = 10$. The most appropriate model is considered to be the one with the lower AIC or BIC value since both these criteria penalize the a large $N_{v}$ number and reward a low objective function. Generally, the Bayesian information criterion is considered the most conservative of the two statistics. Figure 7 shows the AIC and BIC for the “collective” description of the TA gene expression set, i.e. when we describe the complete set of TA-pair with $N = 10*11 = 110$ data points and $\chi ^2$ is the sum of the objective functions of all the TA pairs.

**Figure 8:**
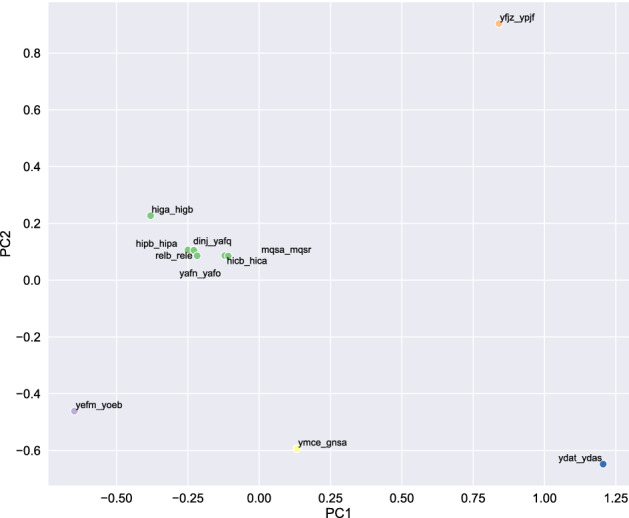
Plot of the largest (*PC*1) vs. the second largest (*PC*2) principal components. A distinction between one main cluster and a set of outliers can be discerned: Central Cluster *dinJ*-*yafQ*, *relB*-*relE*, *yafN*-*yafO*, *higA*-*higB*, *hipB*-*hipA*, *hicB*-*hicA*, and *mqsA*-*mqsR*. Outliers *yefM*-*yoeB*, *ydaT*-*ydaS*, *ymcE*-*gnsA*, and *yfjZ*-*ypjF*.

Finally, it is helpful to compare the values of the constants that we obtained from the minimal ODE model for the different TA pairs. To this end we may view them as a “vector” characterizing the TA pair and we use an unsupervised learning method, namely a Principal Component Analysis (PCA), a statistical procedure that uses an orthogonal transformation to convert a set of observations of possibly correlated variables into a set of values of linearly uncorrelated variables called principal components^61^. PCA is routinely applied to experimental measurments directly for reasons of dimensionality reduction. Using PCA, however, to interpret the parameters of a deterministic ODE model consists a novel approach which has been recently used to interpret the parameters of a fractal kinetics SI model of Covid-19 spreading^62^. Figure 8 shows a plot of the two largest PCA components.

Typically in a PCA plot we try to identify clusters and perceive them as an indication of similar underlying causal behavior. For cluster identification, to avoid subjectivity, we applied a clustering identification algorithm i.e. DBSCAN with parameter $eps = 0.8$^63^. For DBSCAN the number of clusters is not predefined but decided by the algorithm. Here, the clustering algorithm has identified one cluster of 7 TA pairs, namely*dinJ*-*yafQ*, *relB*-*relE*, *yafN*-*yafO*, *higA*-*higB*, *hipB*-*hipA*, *hicB*-*hicA*, and *mqsA*-*mqsR*, which form a large central cluster, and four outliers i.e. the three pairs *yefM*-*yoeB*, *ydaT*-*ydaS*, and *ymcE*-*gnsA*, which have a negative PC2 component, and *yfjZ*-*ypjF* with relatively large PC1 and PC2 values.

In Table 1 we summarize this distinction between a main cluster and several outliers, together with the associated functional classification of the TA pairs. This distinction can serve as a starting point for comparing this statistical result with the wealth of biological information available for each of these TA modules. For the TA module *hipB*-*hipA* for example, the mode of action has been debated over the last years^64,65^, but is still not clear^1^. The similarity of estimated parameters to *higA*-*higB*, *hicB*-*hicA* and other members of the main cluster may be seen as evidence of a functional classification of this TA system as RNA interferases and guide further attempts of functional elucidation, in particular a better understanding of superfamilies of type-II TA systems^66^.

**Table 1:** Identified clusters and their functional classification.

Operon	Functional classification	References
**Main cluster**		
dinJ-yafQ	Ribosome-dependent RNA interferases	^1,67^
relB-relE	Ribosome-dependent RNA interferases	^1,68^
yafN-yafO	Ribosome-dependent RNA interferases	^1,36^
higA-higB	Ribosome-dependent RNA interferases	^1,69^
hipB-hipA	Unknown (involved in persistence)	^1,64^
hicB-hicA	Ribosome-independent RNA interferases	^1,70^
mqsA-mqsR	Ribosome-independent RNA interferases	^1,23^
**Outliers**		
yefM-yoeB	Ribosome-dependent RNA interferases	^1^
ydaT-ydaS	Unknown	^1^
ymcE-gnsA	Inhibitor of phospholipid synthesis	^1,71^
yfjZ-ypjF	Inhibitors of cell division	^1^

Appendix B (Supplementary Materials) contains the results for another time-resolved gene expression data set, namely the data from^72^ which are available at GEO (accession number: GSE131992).

In Appendix C (Supplementary Materials), we present in tabular form the biological information relevant to the members of the clusters identified in Fig. 7 as obtained from The Universal Protein Resource (UniProt), a comprehensive resource for protein sequence and annotation data (https://www.uniprot.org).

## Conclusions

We have proposed a minimal model that is able to capture the dynamics of TA systems in *E. coli* and agrees with experimental high-throughput RNA-Seq data reasonably well. We find that a minimal acceptable model of TA regulation should at least include a negative feedback loop through a TA pair formation and the effect of toxin induced growth modulation. Despite the obvious over-simplifications of the model, e.g. we study each TA pair in isolation, and we do not account for the influence on cell growth due to the remaining toxin proteins, the model is able to replicate a variety of experimental curves.

With the availability of more time-resolved high-quality gene expression data, the description of time courses of systemic components with the help of simple mathematical models can provide an important instrument for the interpretation of such high-throughput data and thus bridge the gap between Theoretical Biology, Statistical Physics and Systems Biology^73^.

## Supplementary information

Supplementary Information.

## Data Availability

The datasets analysed and the custom code used during the current study are available from the corresponding author on reasonable request. They are also available for direct download from Zenodo at 10.5281/zenodo.5162947.
