# Supplementary material for: A minimal model for gene expression dynamics of bacterial type II toxin–antitoxin systems
Source: Sci Rep. 2021 Sep 30;11:19516. doi: 10.1038/s41598-021-98570-z (PMC8484670; doi:10.1038/s41598-021-98570-z)
Supplement: Supplementary file 1 — Supplementary Information. [file 41598_2021_98570_MOESM1_ESM.pdf]

A minimal model for gene expression dynamics of bacterial type II toxin-antitoxin systems

Kosmas Kosmidis[1,*] **and Marc-Thorsten Hütt**[2]

[1]Physics Department, Aristotle University of Thessaloniki, 54124 Thessaloniki, Greece
[2]Department of Life Sciences and Chemistry, Jacobs University Bremen, 28759 Bremen, Germany
[*]kosmask@auth.gr

ABSTRACT

This supplementary materials file contains 3 appendices of the main manuscript "A minimal model for gene expression dynamics of bacterial type II toxin-antitoxin systems"

Appendix A – Estimation of parameters uncertainty

We use stochastic Monte Carlo simulations in order to accurately determine the variances and covariances of the parameter estimates, following the process proposed in[1]. For each TA pair, we generate $Q = 10$ sets of times-series model output data, each set consisting of $N = 5$ stochastic (random) output data points for the toxin and N for the antitoxin, $2N$ times Q data points in total. These points are chosen from a Gaussian random process with zero mean and standard deviation $\sigma_D = 0.1$, superimposed on simulated deterministic model outputs, based on the best estimate of the parameters of the Z-model. We will we the symbol $\hat{p}^0$ for these values of the parameters, which are termed the nominal values in the literature. This simulated stochastic data is then used to obtain a sequence of Q new model parameter estimates $\hat{p}^1, \hat{p}^2, \ldots, \hat{p}^Q$ by fitting the Z-model outputs to the Q sets of stochastic data. The algorithmic steps for the estimation are as follows:

1. We generate Q sets of time-series data, each consisting of N toxin data points and N antitoxin data points, by simulating the noisy outputs $z'_k = z_k\left(\hat{p}^0\right) + e_k, k = 1, \ldots, N$, where e_k are normally distributed random numbers with zero mean and standard deviation $\sigma_D = 0.1$. The $z_k\left(\hat{p}^0\right)$ are the simulated outputs of the (deterministic) Z-model, using the best estimate $\hat{p}^0$ available.

2. We then run the parameter search algorithm for each of the Q sets of simulated data, thereby determining a sequence of Q new parameter estimates $\hat{p}^1, \hat{p}^2, \ldots, \hat{p}^Q$

3. Finally, we determine the sample statistics for the estimated sequence of parameters, i.e. the sample means $\hat{p}$, sample variances $\overline{\sigma}^2$ and sample covariances $\overrightarrow{COV}\left(\hat{p}^j\right)$ for the parameter estimates.

Our results for each TA pair are included in the supplementary materials. We include data for the covariances, correlations and the coefficient of variation $CV = \sigma/\mu$ i.e. the ratio of the standard deviation to the mean for each parameter and each TA pair. Overall, the observed standard deviations for our parameter are within acceptable ranges and those estimates tend to improve significantly, if we increase the number N of the simulated data points, indicating that the Z-model is numerically identifiable[1].

Appendix B – Different environmental and initial conditions

Environmental conditions

We would like to see whether the Z-model is capable to describe the TA gene expression profile for a different high-throuput experiment with different environmental conditions. To this end we use data from[3] which are publically available (GEO accession number: GSE131992). This dataset contains gene expression of *E. coli* at 29 timepoints obtained by switching the culture conditions between starvation and growth. We have used the expression data of ten timepoints from the culture during carbon starvation with time $t = 0$ corresponding to the beginning of the carbon starvation phase. As initial conditions we have used the gene expression data immediately before carbon starvation starts. We have been able to obtain good fits for this dataset also, and, since the parameter space to be investigated is rather large, it is possible that even better accuracy will be achieved under a more exhaustive search. A full analysis of this dataset will be the subject of a subsequent publication. Here, in Fig. 1 we present results for 12 TA pairs, i.e. the 11 pairs of the main document and the mazE-mazF pair whose expression levels are not available in our main dataset.

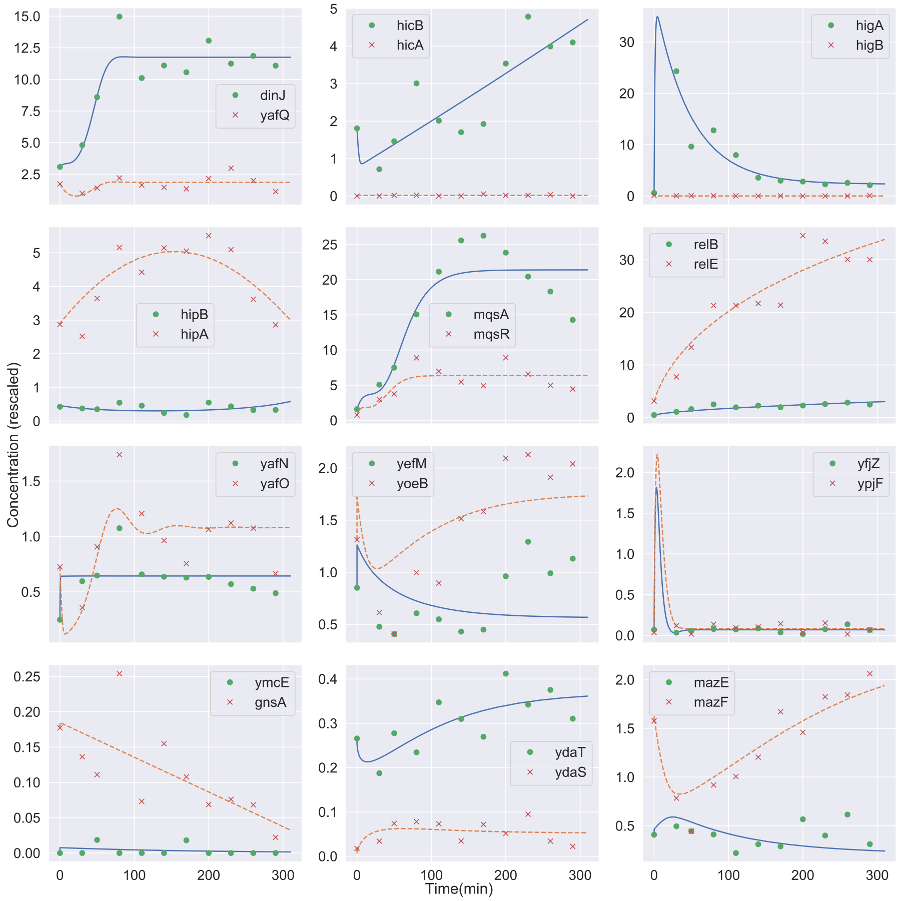

Figure 1. Toxin and antitoxin concentrations as a function of time for 12 TA pairs of *E. coli*. Points represent RNA-Seq data from[3] for antitoxin (circles) and toxin (x-symbols). Solid lines show the result ($z_1(t)$) of the numerical solution of the ODE system, Eqs. (1-3 main document), for the antitoxin. Dashed lines show the corresponding variable ($z_2(t)$) for the toxin.

To compare the values of the constants that we obtained for the different TA pairs under these environmental conditions we apply a Principal Component Analysis (PCA) similar to the one performed for Fig.8 in the main text. For cluster identification, we once again applied a DBSCAN algorithm with parameter $eps = 0.8$[2].

Figure 2 (top) shows a plot of the largest ($PC1$) vs. the second largest ($PC2$) principal components. Again a central cluster is identified comprising 8 TA pairs and four outliers (*mazE-mazF*, *yefM-yoeB*, *ymcE-gnsA* and *hicB-hicA*).

Finally, we have considered analyzing the both experiments together. Thus, we performed PCA on all 11 + 12 TA pairs and plot the result in Fig 2 (bottom). It is intriguing to see that again a central cluster is identified and only three outliers appear, namely *yefM-yoeB* of the first experiment and second experiments, and *hicB-hicA* of the second experiment.

With the model parameters estimated for 11 TA systems in both transcriptome profiles, we can see whether – in spite of the short time series and differences in experimental conditions – the parameter estimates for the same TA pairs are similar between the two datasets. In the PCA plot, this is not apparent, and, indeed, distances in the PCA plane between same TA systems are similar to distances between randomly chosen TA systems.

We expect differences in estimated parameters to mainly come from two sources: (1) functional differences between TA systems, (2) differences in experimental conditions. Furthermore, we expect that some of the model parameters are more sensitive to the first differences, while others are more sensitive to the second.

In order to quantitatively assess this sensitivity, we statistically analyze parameter differences between the two experiments via a proximity network approach: For each parameter P, we connect each TA system from the first experiment with the two closest TA systems from the second experiment, yielding proximity network $G_1(P)$. Next repeat this procedure for the second experiment, connecting each TA system to the two closest TA system from the first experiment, giving proximity network $G_2(P)$. The final proximity network is obtained by taking the union of the node sets and edge sets of $G_1(P)$ and $G_2(P)$, yielding the final proximity network $G(P)$ (which is now symmetrized between the two experiments). The network contains two types of edges, those connecting same TA pairs (type-1 edges) and those connecting different TA systems (type-2 edges). We hypothesize that parameters, for which the proximity network contains unexpectedly many edges of the first type (connecting same TA pairs), are particularly sensitive to differences between TA systems (as often the same TA system has similar parameter values in different data sets). A null model of randomly shuffled TA systems in the edges of the parameter proximity network allows us to compute a z-score for the density of type-1 edges in the proximity network for each parameter. A few cases with z-scores larger than 1 stand out: d_2, d_3 and s_2. Other parameters have z-scores smaller than -1 (and hence seem sensitive to differences in experimental conditions, rather than functional differences between TA systems): s_1, c_0 and b_m.

This sensitivity analysis based on parameter proximity networks, which we introduced here, is from our perspective a convenient way of discriminating these two types of sensitivity.

Different initial conditions

As we mentioned in the main manuscript, setting the initial condition with toxin and anti-toxin concentration to be equal to zero is an arbitrary choice. Here, we are using a different initial condition, i.e. the average concentration across all measurements, which is still an arbitrary but not unreasonable choice and check whether the Z-model is still able to fit the experimental data. We find that the objective function values we get when fitting the data using the above-mentioned choice for the initial conditions are very close to those obtained and presented in the main text for 8 TA pairs out of 11. For the remaining three TA pairs, namely *dinJ-yafQ*, *mqsA-mqsR* and *relB-relE* setting the initial concentration equal to zero leads to considerably lower values of the objective function and, thus, to superior fittings. We cannot, however, exclude the possibility that a more exhaustive search of the parameter space will improve the fitting quality also for these three pairs.

Figure 3 shows the concentrations of toxin and antitoxin for 11 known TA pairs of *E. coli* as a function of time. Symbols represent experimental RNA-Seq data obtained from[4]. The initial concentration for the toxin and antitoxin was set equal to the average concentration across all toxin and antitoxin measurements respectively. Solid lines show the result ($z_1(t)$) of the numerical solution of the ODE system, Eqs. Eqs. (1-3 main document) for the antitoxin. Dashed lines show the corresponding variable ($z_2(t)$) for the toxin. We can confirm that even with a different (yet still arbitrary) choice for the initial concentrations of TA, the Z-model is still able to fit the experimental data.

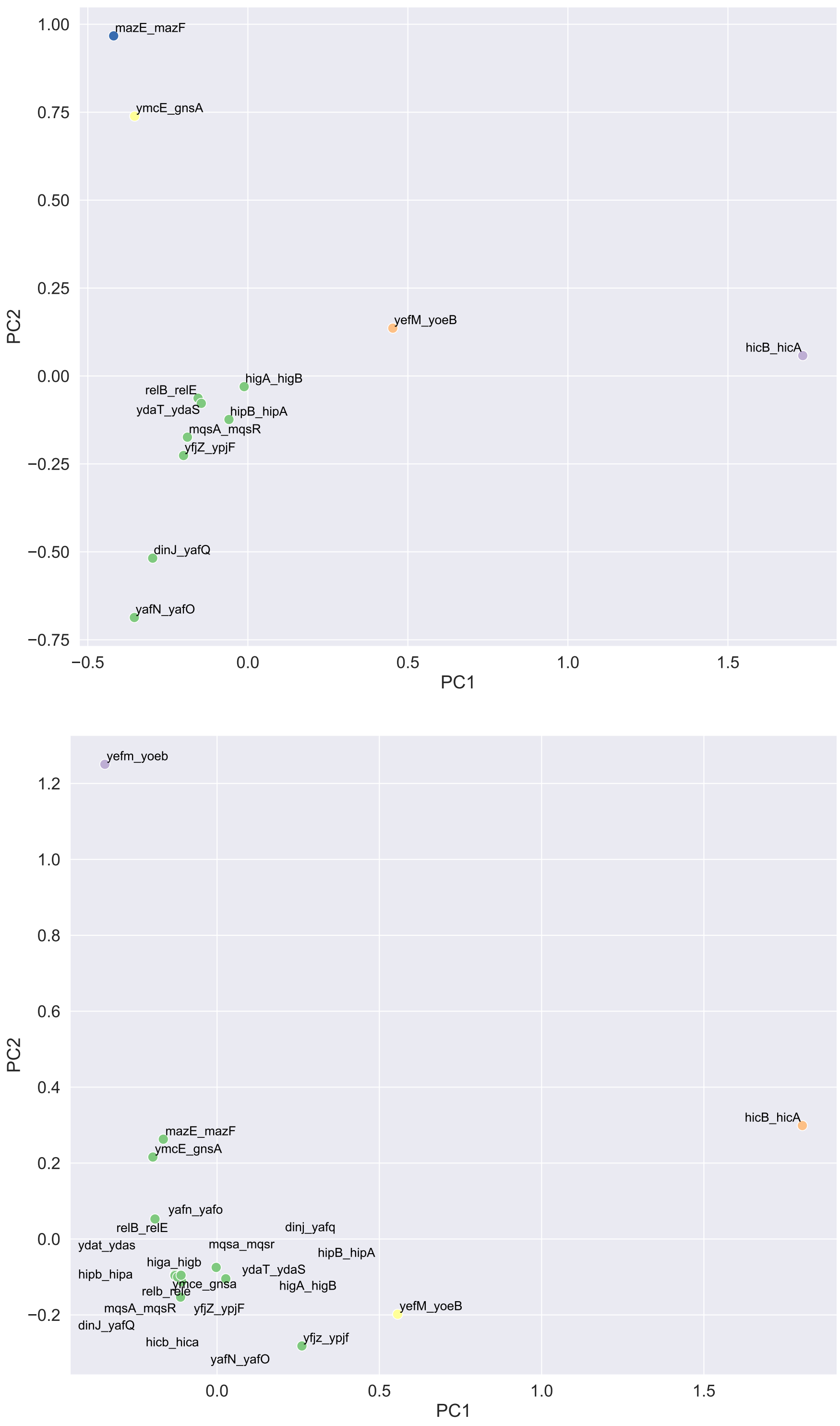

Figure 2. Plot of the largest ($PC1$) vs. the second largest ($PC2$) principal components. Top: Analysis of the second experiment. Bottom: Both experiments analysed as one. Pairs from the first experiment are noted with all letters small (e.g.dinj_yafq) while these of the second with the last letter capitalized (e.g.dinJ_yafQ).

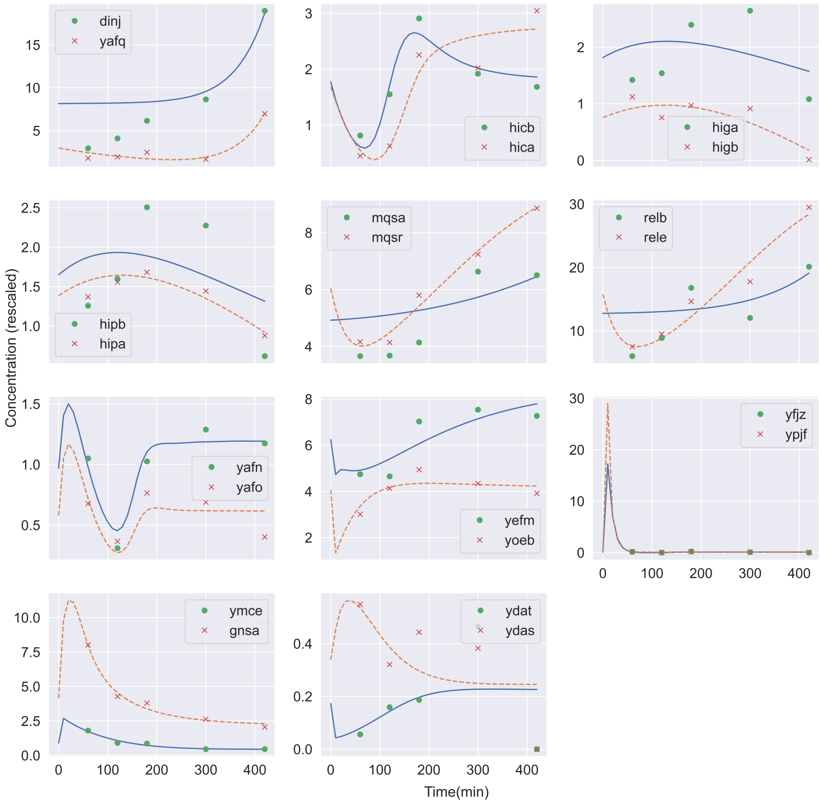

Figure 3. Toxin and antitoxin concentrations as a function of time for 11 known TA pairs of *E. coli*. Points represent RNA-Seq data for antitoxin (circles) and toxin (x-symbols) obtained from[4]. Solid lines show the result $(z_1(t))$ of the numerical solution of the ODE system, Eqs. Eqs. (1-3 main document) for the antitoxin. Dashed lines show the corresponding variable $(z_2(t))$ for the toxin. The initial concentration for the toxin and antitoxin was set equal to the average concentration across all toxin and antitoxin measurements respectively.

Appendix C – Tables

Central Cluster: *dinJ-yafQ*, *relB-relE*, *yafN-yafO*, *higA-higB*, *hipB-hipA*, *hicB-hicA* and *mqsA-mqsR*		
	Antitoxin	Toxin
TA1	Antitoxin *DinJ* Antitoxin component of a type II TA system. A labile antitoxin that counteracts the effect of cognate toxin *yafQ*. *yafQ* and *dinJ* together bind their own promoter, and repress its expression (PubMed:24898247).	mRNA interferase toxin *yafQ* Toxic component of a type II TA system. A sequence-specific mRNA endoribonuclease that inhibits translation elongation and induces bacterial stasis
TA2	Antitoxin *RelB* Antitoxin component of a type II TA system. Counteracts the effect of cognate toxin RelE via direct protein-protein interaction, preventing RelE from entering the ribosome A site and thus inhibiting its endoribonuclease activity.	mRNA interferase toxin *relE* Toxic component of a type II TA system. A sequence-specific, ribosome-dependent mRNA endoribonuclease that inhibits translation during amino acid starvation (the stringent response).
TA3	Antitoxin *yafN* Antitoxin component of a type II TA system. Functions as an mRNA interferase antitoxin; overexpression prevents *yafO* mediated cessation of cell growth and inhibition of cell proliferation.	mRNA interferase toxin *YafO* Toxic component of a type II TA system. A translation-dependent mRNA interferase. Overexpression causes cessation of cell growth and inhibits cell proliferation via inhibition of translation; this blockage is overcome by subsequent expression of antitoxin *yafN*.
TA4	Antitoxin *higA*: Antitoxin component of a type II TA system. Functions as an mRNA interferase antitoxin	mRNA interferase toxin *higB* Toxic component of a type II TA system. A probable translation-dependent mRNA interferase.
TA5	Antitoxin *hipB* Antitoxin component of a type II TA system. Neutralizes the toxic effect of cognate toxin *hipA* Also neutralizes the toxic effect of non-cognate toxin *yjjJ*.	Serine/threonine-protein kinase toxin *hipA* Toxic component of a type II TA system, first identified by mutations that increase production of persister cells, a fraction of cells that are phenotypic variants not killed by antibiotics, which lead to multidrug tolerance. Persistence may be ultimately due to global remodelling of the persister cell's ribosomes.
TA6	Antitoxin *hicB* Antitoxin component of a type II TA system. Functions as an mRNA interferase antitoxin.	Probable mRNA interferase toxin *hicA* Toxic component of a type II TA system. A probable translation-independent mRNA interferase.
TA7	Antitoxin *mqsA* Antitoxin component of a type II TA system. Labile antitoxin that binds to the *mqsR* mRNA interferase toxin and neutralizes its endoribonuclease activity.	mRNA interferase toxin *mqsR* Toxic component of a type II TA system. Plays a significant role in the control of biofilm formation and induction of persister cells in the presence of antibiotics.

Table 1. Central Cluster Toxin-Antitoxin pair description

Outliers		
	Antitoxin	Toxin
TA1	Antitoxin *yefM* Antitoxin component of a type II TA system. Antitoxin that counteracts the effect of the YoeB toxin.*YefM* binds to the promoter region of the *yefM-yoeB* operon to repress transcription, YeoB acts as a corepressor.	Toxin *YoeB* Toxic component of a type II TA system. Its mode of function is controversial; it has been proposed to be an mRNA interferase but also an inhibitor of translation initiation. When overproduced in wild-type cells, inhibits bacterial growth and translation by cleavage of mRNA molecules while it has a weak effect on colony forming ability. Overproduction of Lon protease specifically activates YoeB-dependent mRNA cleavage, leading to lethality.*YefM* binds to the promoter region of the*yefM*-yeoB operon to repress transcription, YeoB acts as a corepressor.
TA2	Antitoxin *ydaT* Antitoxin component of a type II TA system. Neutralizes the toxic effect of cognate toxin *ydaS*	toxin *YdaS* oxic component of a type II TA system
TA3	Antitoxin *ymcE* Antitoxin component of a type II TA system.	Toxin *gnsA* Toxin of a type II TA system.
TA4	Antitoxin *yfjZ* Antitoxin component of a type IV TA system. Antitoxin that counteracts the effect of cognate toxin *YpjF* (PubMed:28257056). Also counteracts the effect of non-cognate toxins CbtA and YfkI	Toxin *YpjF* Toxic component of a type IV TA system. Acts as a dual toxin inhibitor that blocks cell division and cell elongation in genetically separable interactions with FtsZ and MreB

Table 2. Outliers - Toxin-Antitoxin pair description

References

1. DiStefano III J. 2015 *Dynamic systems biology modeling and simulation*. Academic Press.
2. Schubert E, Sander J, Ester M, Kriegel HP, Xu X. 2017 DBSCAN revisited, revisited: why and how you should (still) use DBSCAN. *ACM Transactions on Database Systems (TODS)* **42**, 1–21.
3. Lempp M, Farke N, Kuntz M, Freibert SA, Lill R, Link H. 2019 Systematic identification of metabolites controlling gene expression in E. coli. *Nature communications* **10**, 1–9.
4. Beber ME, Sobetzko P, Muskhelishvili G, Hütt MT. 2016 Interplay of digital and analog control in time-resolved gene expression profiles. *EPJ Nonlinear Biomedical Physics* **4**, 8.
